# An extreme toughening mechanism for soft materials†

**DOI:** 10.1039/d2sm00609j

**Published:** 2022-06-27

**Authors:** Shaoting Lin, Camilo Duque Londono, Dongchang Zheng, Xuanhe Zhao

**Affiliations:** Department of Mechanical Engineering, Massachusetts Institute of Technology Cambridge MA 02139 USA zhaox@mit.edu; Department of Civil and Environmental Engineering, Massachusetts Institute of Technology Cambridge MA 02139 USA

## Abstract

Soft yet tough materials are ubiquitous in nature and everyday life. The ratio between fracture toughness and intrinsic fracture energy of a soft material defines its toughness enhancement. Soft materials’ toughness enhancement has been long attributed to their bulk stress-stretch hysteresis induced by dissipation mechanisms such as Mullins effect and viscoelasticity. With a combination of experiments and theory, here we show that the bulk dissipation mechanisms significantly underestimate the toughness enhancement of soft tough materials. We propose a new mechanism and scaling law to account for extreme toughening of diverse soft materials. We show that the toughness enhancement of soft materials relies on both bulk hysteretic dissipation, and near-crack dissipation due to mechanisms such as polymer-chain entanglement. Unlike the bulk hysteretic dissipation, the near-crack dissipation does not necessarily induce large stress-stretch hysteresis of the bulk material. The extreme toughening mechanism can be potentially universally applied to various soft tough materials, ranging from double-network hydrogels, interpenetrating-network hydrogels, entangled-network hydrogels and slide-ring hydrogels, to unfilled and filled rubbers.

## Abstract 2

This work utilizes a combined effort of experiment and theory to report a new mechanism and scaling law to account for extreme toughening of diverse soft materials.

## Graphical abstract

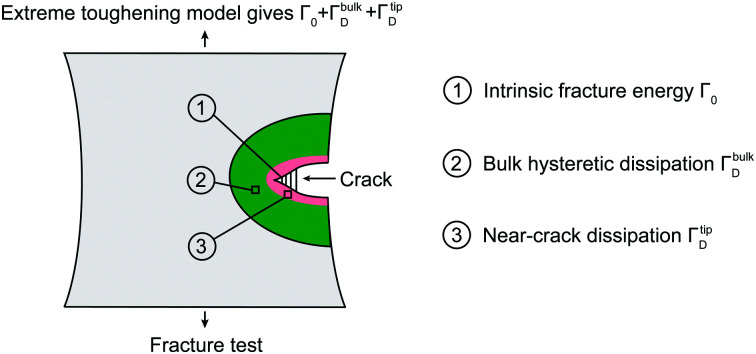

## Introduction

Soft yet tough materials – mainly constituted of polymer networks – are ubiquitous in nature and everyday life, ranging from animal and plant tissues,^1,2^ to synthetic and natural elastomers,^3,4^ to recently developed tough hydrogels including double-network hydrogels,^5,6^ interpenetrating-network hydrogels,^7^ polyampholyte hydrogels,^8^ and slide-ring hydrogels.^9^ For instance, while the Young's moduli of natural muscles,^10^ triple-network elastomers,^11^ and interpenetrating-network hydrogels^7^ are below a few megapascals, their fracture toughness can reach up to 10 000 J m^−2^ – approximating that of tough steels.^12^ Such high fracture toughness of soft materials is crucial for their mechanical integrity and robustness in nature and in engineering applications.

**Fig. 1:**
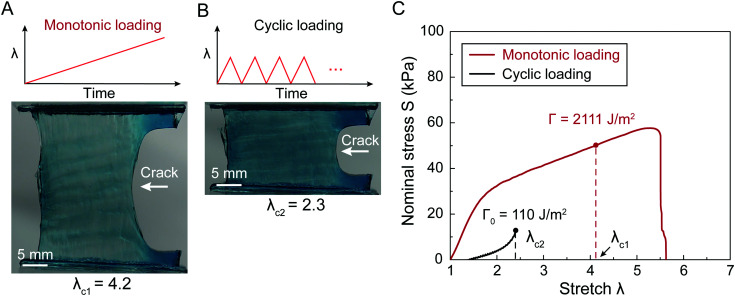
Fracture and fatigue tests of tough hydrogels (with Ca^2+^ and *C*_A_ = 1.0 wt%) measuring fracture toughness *Γ* and fatigue threshold *Γ*_0_. (A) Image of fracture-induced crack extension in a tough hydrogel under monotonic loading, measuring the critical stretch *λ*_c1_ for crack propagation. (B) Image of fatigue-induced crack extension in the same tough hydrogel under cyclic loading, measuring the critical stretch *λ*_c2_ for crack propagation under almost infinite cycles of loading. (C) Nominal stress *S versus* stretch *λ* curves of the un-notched tough-hydrogel samples under monotonic loading and cyclic loading. The un-notched samples have the same material and dimensions as the samples in (A) and (B). Given the identified *λ*_c1_, one can calculate the fracture toughness as 
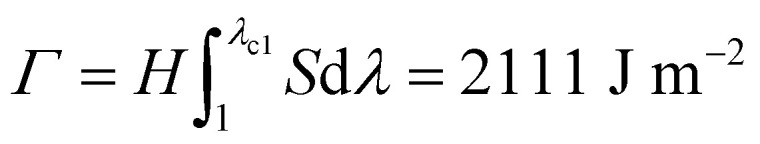
. Given the identified *λ*_c2_, one can calculate the fatigue threshold as 
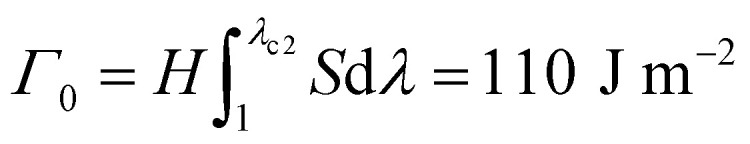
. *H* is the initial height of the sample.

Fracture toughness of soft materials has been long attributed to two physical processes:^13–15^ (1) scission of a layer of polymer chains on the crack tip, and (2) hysteretic mechanical dissipation in the bulk material around the crack tip due to mechanisms such as Mullins effect and viscoelasticity. The first process defines the intrinsic fracture energy *Γ*_0_, and the second process gives the bulk hysteretic dissipation's contribution to fracture toughness *Γ*^bulk^_D_. Consequently, the total fracture toughness of the soft material *Γ*_0_ can be expressed as *Γ* = *Γ*_0_ + *Γ*^bulk^_D_, which is often named the bulk dissipation model.^14,16–20^ The fracture toughness *Γ* of a soft material can be measured as the critical energies required to propagate a crack by a unit area in a material under monotonic loading in a fracture test (Fig. 1(A) and (C)). The fatigue threshold *Γ*_0_ of a soft material can be measured as the critical energy required to propagate a crack by a unit area in a material under infinite cycles of loading in a fatigue test (Fig. 1(B) and (C)). Despite their high fracture toughness up to 10 000 J m^−2^,^3–8^ the measured intrinsic fracture energy of soft materials is usually on the order of 10 to 100 J m^−2^.^18,21^ Soft materials’ toughness enhancement – defined as *Γ*/*Γ*_0_ – has been long attributed to the bulk dissipation mechanisms such as Mullins effect and viscoelasticity.^13–15,17,19,20^

With a combination of experiments and theory, this work shows that the bulk dissipation mechanisms significantly underestimate the toughness enhancement of soft tough materials. We present a new model and scaling law to account for an extreme toughening mechanism in diverse soft tough materials, which relies on both bulk hysteretic dissipation, and near-crack dissipation due to mechanisms such as polymer-chain entanglement and strain-induced crystallization. Using polyacrylamide (PAAm)-alginate hydrogels as an example, we show that the bulk dissipation model underestimates the toughness enhancement of PAAm-alginate hydrogels up to 6.6 times. In contrast, our new model can quantitively predict the toughness enhancement of PAAm-alginate hydrogels across a wide range of bulk hysteresis. We further show that the extreme toughening mechanism can be potentially universally applied to various soft tough materials, ranging from interpenetrating-network hydrogels,^7^ double-network hydrogels,^5,6^ slide-ring gels,^9^ and entangled-network hydrogels,^22,23^ to unfilled and filled rubbers.^24–27^

## Results and discussion

### Extreme toughening model

**Fig. 2:**
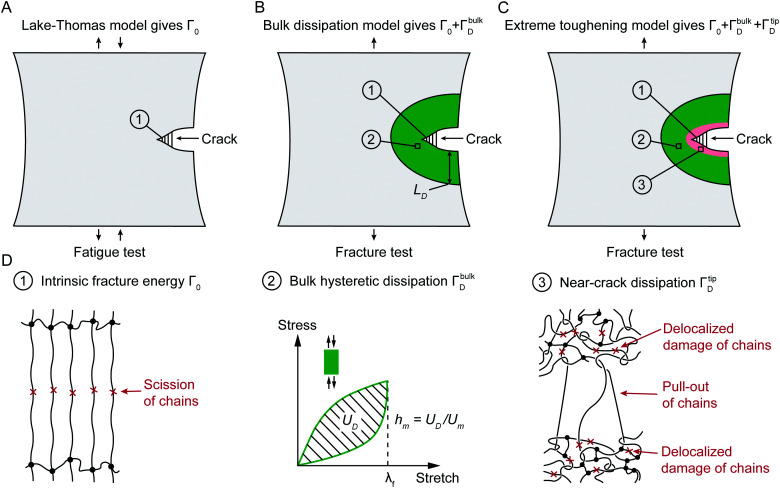
Schematic illustration of molecular mechanisms of three fracture models. (A) Schematic illustration of the Lake–Thomas model accounting for intrinsic fracture energy of soft materials *Γ*_0_. Intrinsic fracture energy of soft materials is typically measured by the fatigue test. (B) Schematic illustration of the bulk dissipation model accounting for two contributions to the total fracture toughness measured in the fracture test: intrinsic fracture energy *Γ*_0_ and bulk hysteretic dissipation *Γ*^bulk^_D_. (C) Schematic illustration of the extreme toughening model accounting for three contributions to the total fracture toughness measured in the fracture test: intrinsic fracture energy *Γ*_0_, bulk hysteretic dissipation *Γ*^bulk^_D_, and near-crack dissipation *Γ*^tip^_D_. (D) Schematic illustration of scission of a layer of chains for the intrinsic fracture energy *Γ*_0_, large stress-stretch hysteresis loop for bulk hysteretic dissipation *Γ*^bulk^_D_, and pull-out of chains and/or delocalized damage of chains for near-crack dissipation *Γ*^tip^_D_.

Fig. 2 schematically illustrates the physical picture of the extreme toughening model. Considering a notched soft material subject to a tensile load, crack propagation in the material first requires the scission of a single layer of polymer chains on the crack path. The required mechanical energy for chain scission divided by the area of crack surface at undeformed state gives the intrinsic fracture energy *Γ*_0_, following Lake–Thomas model (Fig. 2(A) and (D)).^18^ As the crack propagates, the material in a process zone around the crack path experiences a loading–unloading process, which dissipates mechanical energy due to bulk hysteresis, following the bulk dissipation model (Fig. 2(B) and (D)).^14^ The dissipated energy divided by the area of the crack surface at undeformed state contributes to the fracture toughness by *Γ*^bulk^_D_. In addition, if the material contains polymer-chain entanglements, the crack propagation also requires pulling out of chains and delocalized damage of chains adjacent to the crack path, which give the near-crack dissipation (Fig. 2(C) and (D)).^22^ The dissipated energy divided by the area of crack surface at undeformed state further contributes to the fracture toughness by *Γ*^tip^_D_. Therefore, the total fracture toughness of a soft material can be expressed as

1 *Γ* = *Γ*_0_ + *Γ*^bulk^_D_ + *Γ*^tip^_D_

The term *Γ*^bulk^_D_ in eqn (1) can be estimated by

2 *Γ*^bulk^_D_ = *U*_D_*L*_D_

where *U*_D_ is the mechanical energy dissipated per volume of the process zone and *L*_D_ is an effective size of the process zone. *U*_D_ is a measurable quantity defined as 
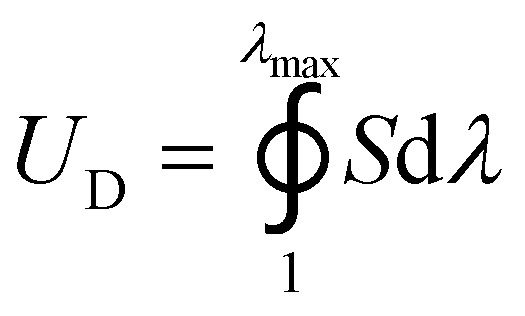
, where *S* and *λ* are stress and stretch of the material under monotonic loading, *λ*_max_ is the maximum stretch at which the material fails under the pure-shear deformation. The effective size of the process zone *L*_D_ can be estimated by the stress distribution profile around the crack tip. As material within the process zone experiences sufficiently high deformation for contributing to bulk hysteretic dissipation, the boundary of the process zone can be determined by identifying a critical length scale.

Without loss of generality, we take the soft material as a neo-Hookean solid. For a neo-Hookean solid under pure-shear fracture test (Fig. 1(A)), the leading order of the nominal stress at a point near the crack tip scales as 
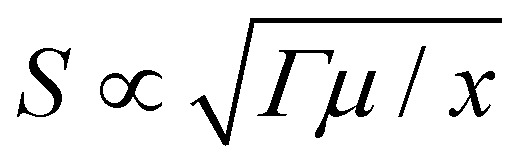
, where *μ* is the shear modulus of the materials and *x* is the distance from the point to the crack tip.^14^ Further, given the maximum nominal stress that the material can reach under the pure-shear deformation is *S*_max_, we can estimate the size of the process zone as

3 *L*_D_ ∝ *Γμ*/*S*_max_^2^ ∝ *Γ*/*U*_max_

where *U*_max_ ∝ *S*_max_^2^/*μ* is the maximum mechanical work done on the material under the pure-shear deformation. A combination of eqn (2) and (3) leads to

4 *Γ*^bulk^_D_ ∝ *Γh*_m_

where *h*_m_ = *U*_D_/*U*_max_ is the maximum stress-stretch hysteresis of the bulk material under the pure-shear deformation. A combination of eqn (1) and (4) further leads to5
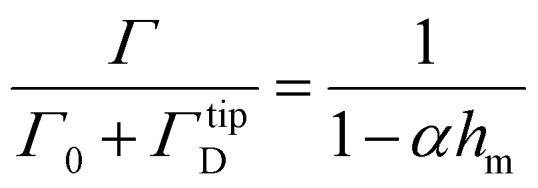
where 0 ≤ *α* ≤ 1 is a dimensionless number depending on the stretch-dependent hysteresis of the bulk materials (*α* = 1 for highly stretchable materials).^14,28^ We further introduce a dimensionless parameter *β* = (*Γ*_0_ + *Γ*^tip^_D_)/*Γ*_0_ ≥ 1 to account for the near-crack dissipation due to chain entanglements. Then, we can derive a governing equation for the toughness enhancement of soft tough materials as6
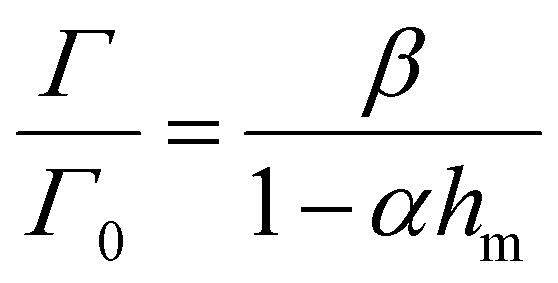
when *h*_m_ = 0, eqn (6) reduces to *Γ*/*Γ*_0_ = *β*, corresponding to the toughening of soft materials by the near-crack dissipation. When *β* = 1, eqn (6) reduces to *Γ* + *Γ*_0_ = 1/(1 − *αh*_m_), which recovers the bulk dissipation model.

### Experiments

We chose polyacrylamide-alginate (PAAm-alginate) hydrogels as a model material to validate the model. Due to its extremely high fracture toughness, PAAm-alginate hydrogel has been intensively exploited as a key component for devices and machines with examples such as tough hydrogel bonding,^29^ soft robots,^26^ hydrogel bandage,^30^ acoustic metamaterials,^31^ ultrasound imaging,^32^ and living sensors.^33^ A PAAm-alginate hydrogel is made of two interpenetrating polymer networks: covalently crosslinked long-chain PAAm network, and ionically-crosslinked short-chain alginate network. The covalently crosslinked long-chain PAAm network provides the material's stretchable elasticity, and the ionically crosslinked short-chain alginate network dissociates as the material is highly deformed, giving the material's bulk hysteresis. In this work, we maintain the concentration and crosslinking density of PAAm network while varying the concentration and crosslinking density of alginate network, thereby tuning the bulk hysteresis of PAAm-alginate hydrogels.

**Fig. 3:**
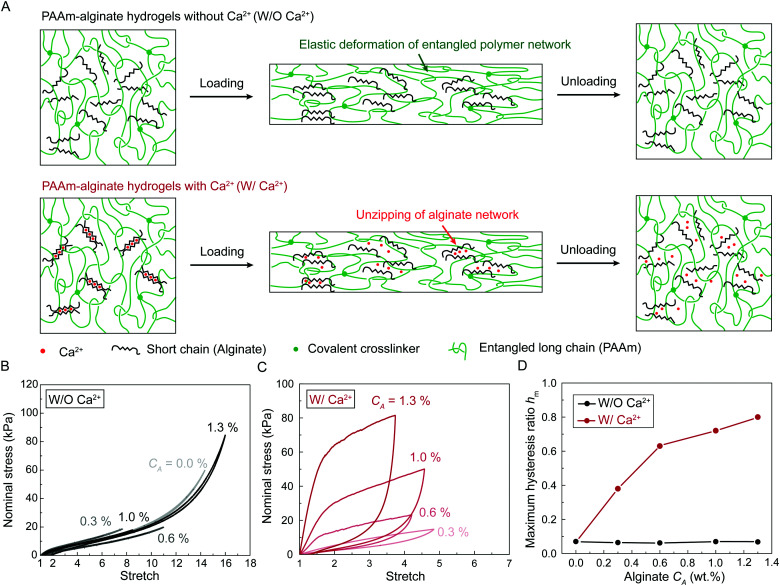
Stress-stretch hysteresis in hydrogels with and without Ca^2+^. (A) Schematic illustration of molecular pictures of the two series of PAAm-alginate hydrogels under a single cycle of loading and unloading. (B) Nominal stress *versus* stretch curves of hydrogels without Ca^2+^ containing various alginate concentration *C*_A_ under a single cycle of loading and unloading. (C) Nominal stress *versus* stretch curves of hydrogels with Ca^2+^ containing various alginate concentration *C*_A_ under a single cycle of loading and unloading. (D) The maximum hysteresis *h*_m_ as a function of alginate concentration *C*_A_ for the two series of PAAm-alginate hydrogels.

We synthesize two series of PAAm-alginate hydrogels (Fig. 3(A)). As schematically illustrated in Fig. 3(A), both series of PAAm-alginate hydrogels contain covalently crosslinked PAAm long-chain network. In our recent work, we varied the average number of monomers between neighboring crosslinkers by changing crosslinker densities while maintaining the polymer content in the polyacrylamide (PAAm) hydrogels to systematically control the level of chain entanglement. In this work, the average number of AAm monomers between neighboring crosslinkers in the as-prepared state is fixed as *N* = 2263, giving substantial chain entanglement according to our rheology characterization.^22^ In the first series of PAAm-alginate hydrogels, the sodium alginate polymers are uncrosslinked mobile chains. In the second series of PAAm-alginate hydrogels, the sodium alginate polymers are ionically crosslinked into polymer networks. Unless otherwise stated, we denote the first series of PAAm-alginate hydrogels as hydrogels without Ca^2+^ (*i.e.*, W/O Ca^2+^), and denote second series of PAAm-alginate hydrogels as hydrogels with Ca^2+^ (*i.e.*, W/Ca^2+^).

We first characterize the stress-stretch curves of the two series of PAAm-alginate hydrogels up to failure points under the pure-shear deformation. For hydrogels without Ca^2+^, the sodium alginate concentration *C*_A_ has little effect on the nonlinear stress-stretch relationship (Fig. S1, ESI†), because the alginate chains are uncrosslinked mobile chains and do not contribute to the elasticity of the hydrogels. In contrast, for hydrogels with Ca^2+^, the sodium alginate concentration has significant impacts on stress-stretch curves (Fig. S1, ESI†). As the sodium alginate concentration *C*_A_ increases, the nominal stress increases accordingly while the ultimate stretch remains constant. Compared to hydrogels without Ca^2+^, the ultimate stretches of hydrogels with Ca^2+^ decrease drastically, possibly because the ionically crosslinked alginate network suppresses the stretchablity of the polyacrylamide network.

We further characterize the stress-stretch hysteresis of the two series of PAAm-alginate hydrogels. Fig. S2 (ESI†) plots the stress-stretch curves under one cycle of loading at different stretch levels for hydrogels without Ca^2+^. The measured bulk hysteresis is consistently below 10% even when the maximum stretch approaches the failure points (Fig. 3(B) and (D)). This is because the uncrosslinked alginate polymers do not contribute to elasticity or hysteresis of the material and the entangled PAAm polymer network exhibits low bulk hysteresis.^22^ In contrast, since alginate polymers form the ionically crosslinked network in hydrogels with Ca^2+^, the alginate network unzips progressively when the material is highly deformed, which gives the huge bulk hysteresis (Fig. 3(A)). Fig. 3(C) and Fig. S3 (ESI†) plot the stress-stretch curves under one cycle of loading at different stretch levels for hydrogels with Ca^2+^. The bulk hysteresis of hydrogels with Ca^2+^ monotonically increases with the applied stretch and reaches a maximum plateau. We take the maximum plateau as the maximum bulk hysteresis *h*_m_. As summarized in Fig. 3(D), the maximum bulk hysteresis of hydrogels with Ca^2+^ increases with the alginate concentration *C*_A_. This further indicates the critical role of ionically crosslinked alginate network in promoting the bulk hysteresis.

We next use fracture and fatigue tests to measure the fracture toughness *Γ* and fatigue threshold *Γ*_0_ of the two series of PAAm-alginate hydrogels. We first adopt both pure-shear and single-notch methods to measure their fatigue thresholds (Fig. S4–S6, ESI†), which give their intrinsic fracture energies *Γ*_0_. The measured fatigue thresholds of both series of hydrogels are consistently around 110 J m^−2^. (Unless otherwise stated, the reported values of fatigue threshold have been converted to the corresponding values in the as-prepared or reference state by accounting for swelling of the hydrogels. The swelling ratios in volume are summarized in Fig. S7, ESI.†) This indicates the presence of ionically crosslinked alginate network does not contribute to the fatigue threshold (Fig. 3(B)), because the resistance to fatigue crack propagation after prolonged cycles of loading is the energy required to fracture a layer of PAAm polymer chains (*i.e.*, the intrinsic fracture energy), which is unaffected by the additional bulk dissipation mechanisms by unzipping the ionically crosslinked alginate network.^34^

We further use the pure-shear method to measure the fracture toughness of the two series of PAAm-alginate hydrogels. For hydrogels without Ca^2+^, the alginate concentration *C*_A_ has little effect on the fracture toughness (Fig. S8, ESI†). Even though the bulk hysteretic dissipations in hydrogels without Ca^2+^ are negligible, the measured fracture toughness is still relatively high (480 J m^−2^), about 4.3 times of their fatigue threshold (*i.e.*, 110 J m^−2^). This indicates that the difference between fracture toughness and fatigue threshold of hydrogels without Ca^2+^ is due to the near-crack dissipation, not the bulk dissipation.^22^ Therefore, the fracture toughness of hydrogels without Ca^2+^ measures *Γ*_0_ + *Γ*^tip^_D_ (Fig. 3). Our recent work has systematically studied the presence of chain entanglement as a new toughening mechanism. Once a crack propagates in an entangled polymer network, the highly entangled polymer chains across the crack plane are pulled out, potentially dissipating substantial energy due to abundant intermolecular interactions between neighboring chains. In addition, once the entangled chains around the crack tip are highly stretched, scissions of chains can be delocalized to multiple adjacent layers around the crack plane, dissipating more energy than fracturing a single layer of chains.

**Fig. 4:**
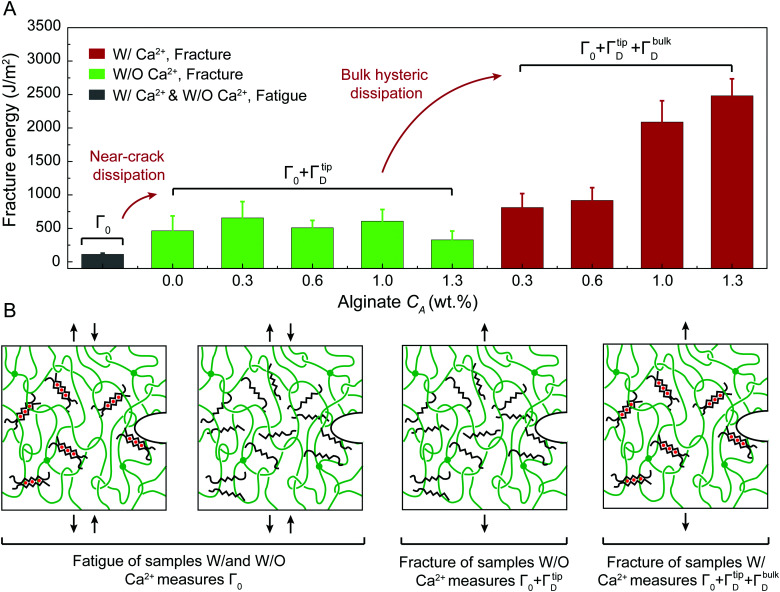
Summarized fracture toughness and fatigue threshold of hydrogels with and without Ca^2+^. (A) Three levels of fracture energies of the two series of PAAm-alginate hydrogels. (B) Schematic illustration of fatigue test of hydrogels with and without Ca^2+^ measuring *Γ*_0_, fracture test of hydrogels without Ca^2+^ measuring *Γ*_0_ + *Γ*^tip^_D_, and fracture test of hydrogels with Ca^2+^ measuring *Γ*_0_ + *Γ*^bulk^_D_ + *Γ*^tip^_D_.

For hydrogels with Ca^2+^, the alginate concentration *C*_A_ significantly affects the fracture toughness (Fig. 4(A) and Fig. S8, ESI†). As *C*_A_ increases, the fracture toughness of hydrogels with Ca^2+^ increases drastically from 500 to 2800 J m^−2^ (Fig. 4(A)). This enhancement of the fracture toughness is due to the bulk hysteretic dissipation by unzipping the ionically crosslinked alginate network; the level of bulk hysteretic dissipation is determined by the alginate concentration *C*_A_. Consequently, the fracture toughness of hydrogels with Ca^2+^ measures *Γ*_0_ + *Γ*^bulk^_D_ + *Γ*^tip^_D_ (Fig. 4).

### Comparison between experiments and models

**Fig. 5:**
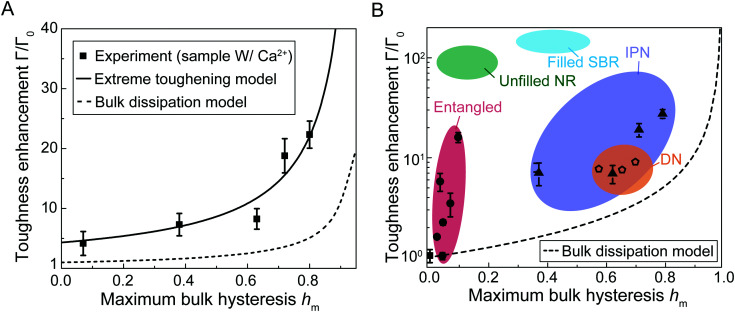
Comparisons between experiments and models for toughness enhancement *versus* maximum bulk hysteresis *h*_m_. (A) Comparisons of toughness enhancement *Γ*/*Γ*_0_*versus* maximum bulk hysteresis *h*_m_ between the experimental results and the two models (extreme toughening model and bulk dissipation model). (B) Toughness enhancement *Γ*/*Γ*_0_ and maximum bulk hysteresis *h*_m_ for various soft tough materials including interpenetrating-network (IPN) hydrogels,^7,21^ double-network (DN) hydrogels,^5,35^ entangled hydrogels,^22^ unfilled natural rubbers (NR),^24,25^ and filled styrene-butadiene rubbers (SBR).^26,27^ The bulk dissipation model consistently underestimates the toughness enhancement of these soft tough materials.

Given the measured maximum bulk hysteresis *h*_m_, fracture toughness *Γ*, and fatigue threshold *Γ*_0_ of the hydrogels with Ca^2+^, we summarize the measured toughness enhancement *Γ*/*Γ*_0_ as a function of the measured maximum bulk hysteresis *h*_m_ in Fig. 5(A). When the maximum bulk hysteresis is small, the toughness enhancement can still achieve 4.3. As the maximum bulk hysteresis increases, the toughness enhancement increases accordingly. When the maximum bulk hysteresis reaches 80%, the toughness enhancement can be as high as 22.

We then use eqn (6) to calculate the relationship between fracture toughness enhancement *Γ*/*Γ*_0_ and maximum bulk hysteresis *h*_m_. The parameter *β* = (*Γ*_0_ + *Γ*^tip^_D_)/*Γ*_0_ is identified as 4.3 given the measured *Γ*_0_ + *Γ*^tip^_D_ (*i.e.*, 480 J m^−2^) and the measured *Γ*_0_ (*i.e.*, 112 J m^−2^). The parameter *α* is taken as 1 since PAAm-alginate hydrogels are highly stretchable. Given the identified *β* and *α*, we can plot toughness enhancement *Γ*/*Γ*_0_ as a function of the maximum bulk hysteresis *h*_m_. As shown in Fig. 5(A), our extreme toughening model can quantitatively capture the toughness enhancement across a wide range of the maximum bulk hysteresis *h*_m_. In contrast, we also plot *Γ*/*Γ*_0_*versus h*_m_ following the bulk dissipation model, and the predicted toughness enhancement is significantly lower than the experimental results.

We further summarize reported toughness enhancement and maximum bulk hysteresis of various soft tough materials, including interpenetrating-network hydrogels,^7,21^ double-network hydrogels,^5,35^ entangled-network hydrogels,^22^ slide-ring gels,^9^ unfilled natural rubbers,^24,25^ and filled styrene-butadiene rubbers.^26,27^ The predicted toughness enhancements following the bulk dissipation model are consistently lower than the measured values (Fig. 5(B)). For example, the toughness enhancement of the interpenetrating-network hydrogels^7^ with bulk hysteresis of around 80% should be around 5 following the bulk dissipation model, but the measured toughness enhancement is more than 20.^21^ The toughness enhancement of the double-network hydrogels^5^ with bulk hysteresis of around 70% should be around 3.3 following the bulk dissipation model, but the measured toughness enhancement is at least 8.^35^ The toughness enhancement of unfilled natural rubbers with bulk hysteresis of around 20% should be around 1.2 following the bulk dissipation model,^25^ but the measured toughness enhancement is as high as 100.^24^ We envision our extreme toughening model can quantitatively capture the toughness enhancements of various soft toughen materials, because nearly all these soft tough materials contain substantial near-crack dissipation due to mechanisms such as chain entanglements.

## Discussion

Here, we use a combination of experiments and theory to show that the bulk dissipation mechanisms significantly underestimate the toughness enhancement of soft tough materials. We propose a new mechanism and scaling law to account for the extreme toughening of diverse soft tough materials. The extreme toughening relies on both bulk hysteretic dissipation and near-crack dissipation due to mechanisms such as polymer-chain entanglement and strain-induced crystallization. Using polyacrylamide (PAAm)-alginate hydrogels as an example, we show that the bulk dissipation model underestimates the toughness enhancement of PAAm-alginate hydrogels up to 6.6 times. In contrast, our new extreme toughening model can quantitively predict the toughness enhancement of PAAm-alginate hydrogels across a wide range of bulk hysteresis. We envision the extreme toughening mechanism can be potentially universally applied to various soft tough materials, ranging from double-network hydrogels, interpenetrating-network hydrogels, entangled-network hydrogels and slide-ring hydrogels, to unfilled and filled rubbers. Our study resolves a fundamental dilemma in toughening mechanisms of soft materials. It is hoped that this work can help the development of next-generation tough, fatigue-resistant, and resilient soft materials. One limitation of the current study is there is no quantitative differentiation of the process zone size for bulk hysteretic dissipation and the process zone size for near-crack dissipation. Future work will focus on new experimental tools to visualize and quantify the damage occurring in the process zones due to bulk hysteretic dissipation and near-crack dissipation.

## Author contributions

Shaoting Lin: conceptualization, methodology, data curation, formal analysis, investigation, writing – original draft, writing – review & editing. Camilo Duque Londono: data curation, formal analysis, writing – review & editing. Dongchang Zheng: data curation, formal analysis. Xuanhe Zhao: conceptualization, methodology, formal analysis, investigation, writing – original draft, writing – review & editing.

## Conflicts of interest

The authors declare no competing interest.

## Supplementary Material

SM-018-D2SM00609J-s001
