# Supplementary material for: An extreme toughening mechanism for soft materials
Source: Soft Matter. 2022 Jun 27;18(31):5742–9. doi: 10.1039/d2sm00609j (PMC9364334; doi:10.1039/d2sm00609j)
Supplement: SM-018-D2SM00609J-s001 [file SM-018-D2SM00609J-s001.pdf]

Electronic Supplementary Material (ESI) for Soft Matter.
This journal is © The Royal Society of Chemistry 2022

Supplementary Information for

An Extreme Toughening Mechanism for Soft Materials

Shaoting Lin[1], Camilo Duque Londono[1], Dongchang Zheng[1,2], Xuanhe Zhao[1,3*]

[1]Department of Mechanical Engineering, Massachusetts Institute of Technology, Cambridge, MA 02139, USA.

[2]CAS Key Laboratory of Mechanical Behavior and Design of Materials, Department of Modern Mechanics, University of Science and Technology of China, Hefei 230026, People's Republic of China

[3]Department of Civil and Environmental Engineering, Massachusetts Institute of Technology, Cambridge, MA 02139, USA

*Xuanhe Zhao

Email: zhaox@mit.edu

This PDF file includes:

Supplementary text

Figures S1 to S8

Supplementary Information Text

Material Synthesis. Unless otherwise specified, the chemicals used in this work were purchased from Sigma-Aldrich and used without further modification. To synthesize the two series of PAAm-alginate hydrogels, we dissolved the powders of sodium alginate (Sigma-Aldrich A2033) and acrylamide ($M_W = 71\ g/mol$, Sigma-Aldrich A8887) in deionized water, yielding pre-gel solution with fixed acrylamide concentration at 12 wt% but varied alginate concentration from 0.0, 0.3, 0.6, 1.0, to 1.3 wt%. For each batch of sample, we further added 110 µL 0.1 M ammonium persulfate (APS, Sigma-Aldrich A3678) as the thermal-initiator, 500 µl 0.23 wt% N,N'-Methylenebisacrylamide (MBAA, $M_W = 154\ g/mol$, Sigma-Aldrich 146072) as the crosslinker, and 20 µL N,N,N',N'-tetramethylethylenediamine (TEMED, Sigma-Aldrich T9281) as the crosslinking accelerator in 10 mL of the pre-gel solution. Thereafter, we poured the solution into a customized acrylic mold measuring 34 × 6 × 1.5 mm^3. The mold was placed in a 50 °C oven to complete the thermal-induced free radical polymerization, resulting in the first series of PAAm-alginate hydrogels. The cured samples were further soaked in a bath of 0.01 wt% calcium chloride (Sigma-Aldrich C4901) solution for 24 hours to form ionic crosslinking between Ca^{2+} and alginate chains, resulting in the second series of PAAm-alginate hydrogels.

Chemically anchoring PAAm on glass fixtures. To accurately capture nonlinear stress-stretch response of hydrogels up to material failures, we chemically anchored PAAm on glass fixtures. The glass slides were treated by oxygen plasma (30 W at a pressure of 400 mtorr, Harrick Plasma PDC-001) for 2 min. During oxygen plasma treatment, silicon oxide layers on glass slides react to hydrophilic hydroxyl groups by oxygen radicals produced by the oxygen plasma. After the plasma treatment, we immersed the glass slides in a bath of silane solution for 1 hour, which was prepared by mixing 500 mL deionized water, 50 µL of acetic acid with pH 3.5, and 2 mL silane 3-(trimethoxysilyl) propyl methacrylate (TMSPMA, Sigma-Aldrich 440159). The hydroxyl groups produced by oxygen plasma form hydrogen bonds with silanes in the silane solution. Thereafter, glass slides were washed with deionized water and dried using nitrogen gas. The silane treated glass slides were placed in the customized mold for sealing the pre-gel solution. During the curing of the pre-gel solution, a copolymerization also occurs between the methacrylate group in the grafted TMSPMA and the acrylate groups in acrylamide under a thermal-induced free radical polymerization. Consequently, long-chain PAAm polymer network was covalently anchored to

the glass fixtures, so that we can capture the nonlinear large deformation of hydrogels up to failure of materials rather than failure at the interface between materials and fixtures.

Measurement of stress-stretch curves and bulk hysteresis. We used mechanical tester (Zwick/Roell Z2.5) to measure the stress-stretch curves of hydrogels in a rectangular shape at a fixed loading speed of 10 mm/min. The nominal stress S was measured from the recorded force divided by width and thickness of the sample, which measures 40 mm and 1.5 mm, respectively. The applied stretch λ was monitored by the recorded displacement divided by height of the sample, which measures 10 mm. We first performed tensile loading under a monotonic loading, measuring the stress-stretch curves of hydrogels up to the failure of material. We then performed a single cycle of loading and unloading on the other pristine sample with controlled maximum applied stretch λ_{applied} from a relatively small value to a high value approaching the ultimate stretch of the material. The bulk hysteresis was calculated by the ratio of the enclosed loop area of loading and unloading stress-stretch curves to the enclosed area of loading curves, namely, $h\left(\lambda_{\text{applied}}\right) = \oint^{\lambda_{\text{applied}}} Sd\lambda / \int_1^{\lambda_{\text{applied}}} Sd\lambda$. The bulk hysteresis typically increases monotonically with λ_{applied}, and reaches a maximum plateau value, which was identified as the maximum bulk hysteresis h_m.

Fracture test. We adopted pure-shear tensile method to measure the fracture energies of the two series of PAAm-alginate hydrogels (Fig. S8a). Given the measured nominal stress versus stretch curves, we can calculate the mechanical work done on the unnotched sample as $U\left(\lambda_{\text{applied}}\right) = \int_1^{\lambda_{\text{applied}}} Sd\lambda$. We further introduced a sharp crack in the other pristine sample with the identical dimension of the unnotched sample (i.e., width of 40 mm, thickness of 10 mm, and height of 1.5 mm). The crack length was controlled about one fourth of the width of the sample. We then applied tensile loading on the notched sample at a fixed loading speed of 10 mm/min, measuring a critical stretch λ_c, at which crack propagates steadily. The measured critical stretches are summarized in Fig. S8b. Given the measured λ_c, we can calculate the fracture toughness as $\Gamma = H\int_1^{\lambda_c} Sd\lambda$. The measured fracture energies are summarized in Fig. S8c.

Fatigue test. We adopted both pure-shear and single-notch methods to measure the fatigue thresholds of the two series of PAAm-alginate hydrogels. For the pure-shear method, we fabricated

the sample into a rectangular shape with dimensions of 34 × 6 × 1.5 mm^3 at its as-prepared state. The sample was chemically anchored on glass fixtures. As schematically illustrated in Fig. S5a, we first cyclically load an unnotched sample to measure the steady-state nominal stress versus stretch curve under cyclic loading. The strain energy density W of the unnotched sample under the Nth cycle of maximum applied stretch of λ^{A} can be calculated as $W\left(\lambda^{\mathrm{A}}, N\right)=\int_{1}^{\lambda^{\mathrm{A}}} S(N) d\lambda$ with S and λ being the steady-state nominal stress and stretch, respectively. Thereafter, a cyclic loading with the same maximum stretch of λ^{A} is applied on the other notched sample with the same dimensions as the unnotched sample. The corresponding applied energy release rate can be calculated as $G\left(\lambda^{A}, N\right)=H\int_{1}^{\lambda^{\mathrm{A}}} S(N) d\lambda$ with H being height of the sample. We used a camera (Imaging Source, 30 μm/pixel) to record the crack extension (i.e., ΔC) over cycles, measuring the crack extension rate dC/dN. By systematically varying the applied stretch λ^{A}, we can obtain a plot of dC/dN versus G. Figure S4b shows a representative plot of dC/dN versus G for the hydrogel with Ca^{2+} and alginate concentration of C_A = 1.0 wt%. By linearly extrapolating the curve of dC/dN versus G to the intercept with the abscissa, one can approximately identify the measured fatigue threshold Γ_0 in the swollen state. Give the swelling ratio in volume λ_V, one can further calculate the fatigue threshold of the hydrogel in the as-prepared state by $\lambda_V^{2/3}\Gamma_0$ (Fig. S7).

For the single-notch method, we fabricated the sample into a dog-bone shape. As schematically illustrated in Fig. S5a, we first cyclically load an unnotched sample to measure the steady-state nominal stress versus stretch curve under cyclic loading. The strain energy density W of the unnotched sample under the Nth cycle of maximum applied stretch of λ^{A} can be calculated as $W\left(\lambda^{\mathrm{A}}, N\right)=\int_{1}^{\lambda^{\mathrm{A}}} S(N) d\lambda$ with S and λ being the steady-state nominal stress and stretch, respectively. Thereafter, the same cyclic stretch λ^{A} is applied on the notched sample, measuring the evolution of the cut length in undeformed state c as a function of the cycle number. The initial cut length is smaller than one fifth the width of the sample. The applied energy release rate can be calculated as $G\left(\lambda^{A}, N\right)=2k\left(\lambda^{A}\right)c(N)\int_{1}^{\lambda^{\mathrm{A}}} S(N) d\lambda$, where $k=3/\sqrt{\lambda^{A}}$ and c is the current crack length at undeformed configuration. We used a camera (Imaging Source, 30 μm/pixel) to record the crack extension (i.e., ΔC) over cycles, measuring the crack extension rate dC/dN. By

systematically varying the applied stretch λ^{A}, we can obtain a plot of dC/dN versus G. Figure S5c shows a representative plot of dC/dN versus G for the hydrogel with Ca^{2+} and alginate concentration of C_A = 1.0 wt%. By linearly extrapolating the curve of dC/dN versus G to the intercept with the abscissa, one can approximately identify the measured fatigue threshold Γ_0 in the swollen state. Give the swelling ratio in volume λ_V, one can further calculate the fatigue threshold of the hydrogel in the as-prepared state by $\lambda_V^{2/3}\Gamma_0$ (Fig. S7).

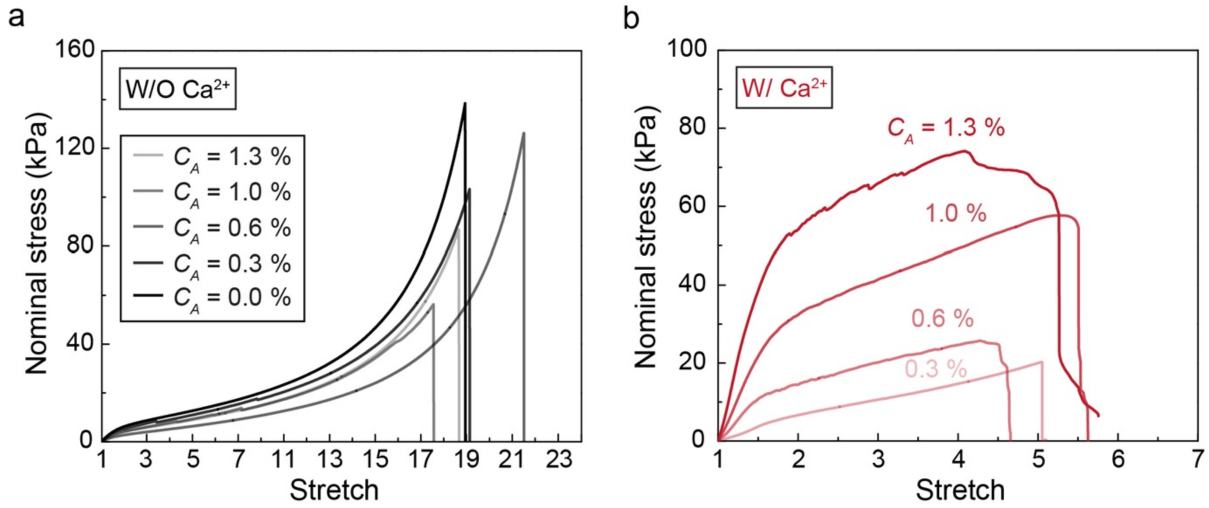

Fig. S1. Nominal stress versus stretch curves of (a) PAAm-alginate hydrogels without Ca^{2+} containing various alginate concentrations C_A and (b) PAAm-alginate hydrogels with Ca^{2+} containing various alginate concentrations C_A.

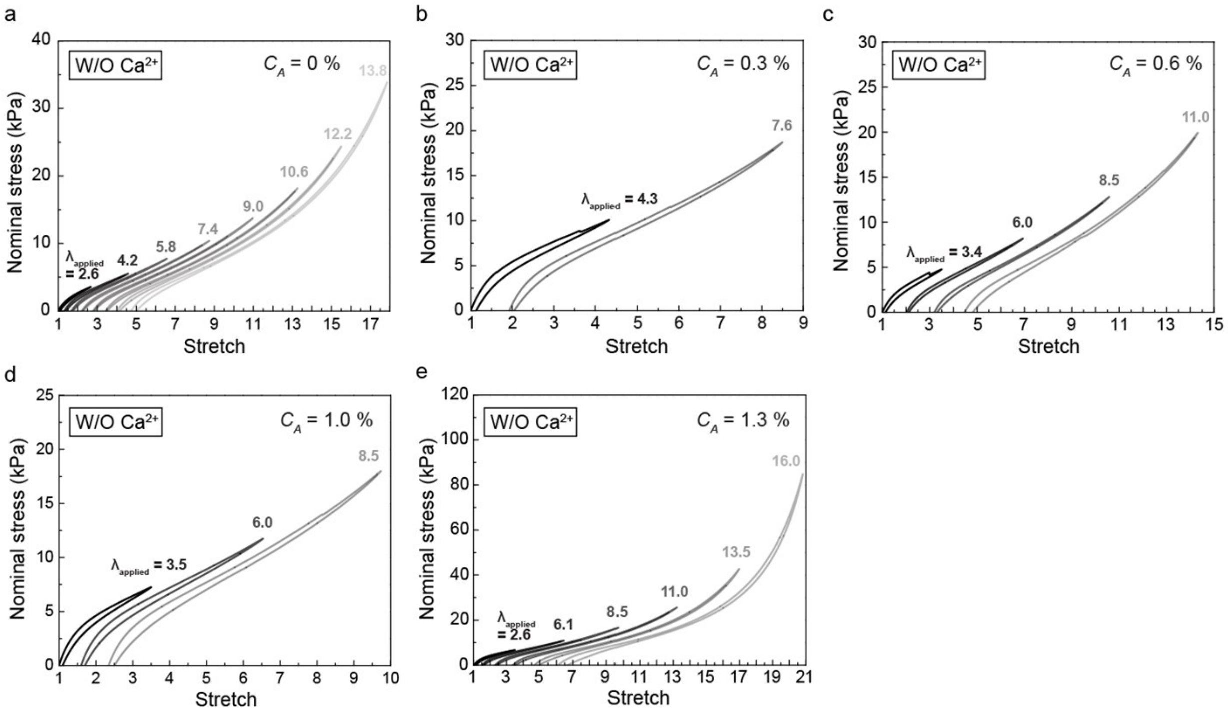

Fig. S2. Nominal stress versus stretch curves of PAAm-alginate hydrogels without Ca^{2+} under one cycle of loading and unloading. (a) C_A = 0.0 wt%. (b) C_A = 0.3 wt%. (c) C_A = 0.6 wt%. (d) C_A = 1.0 wt%. (e) C_A = 1.3 wt%.

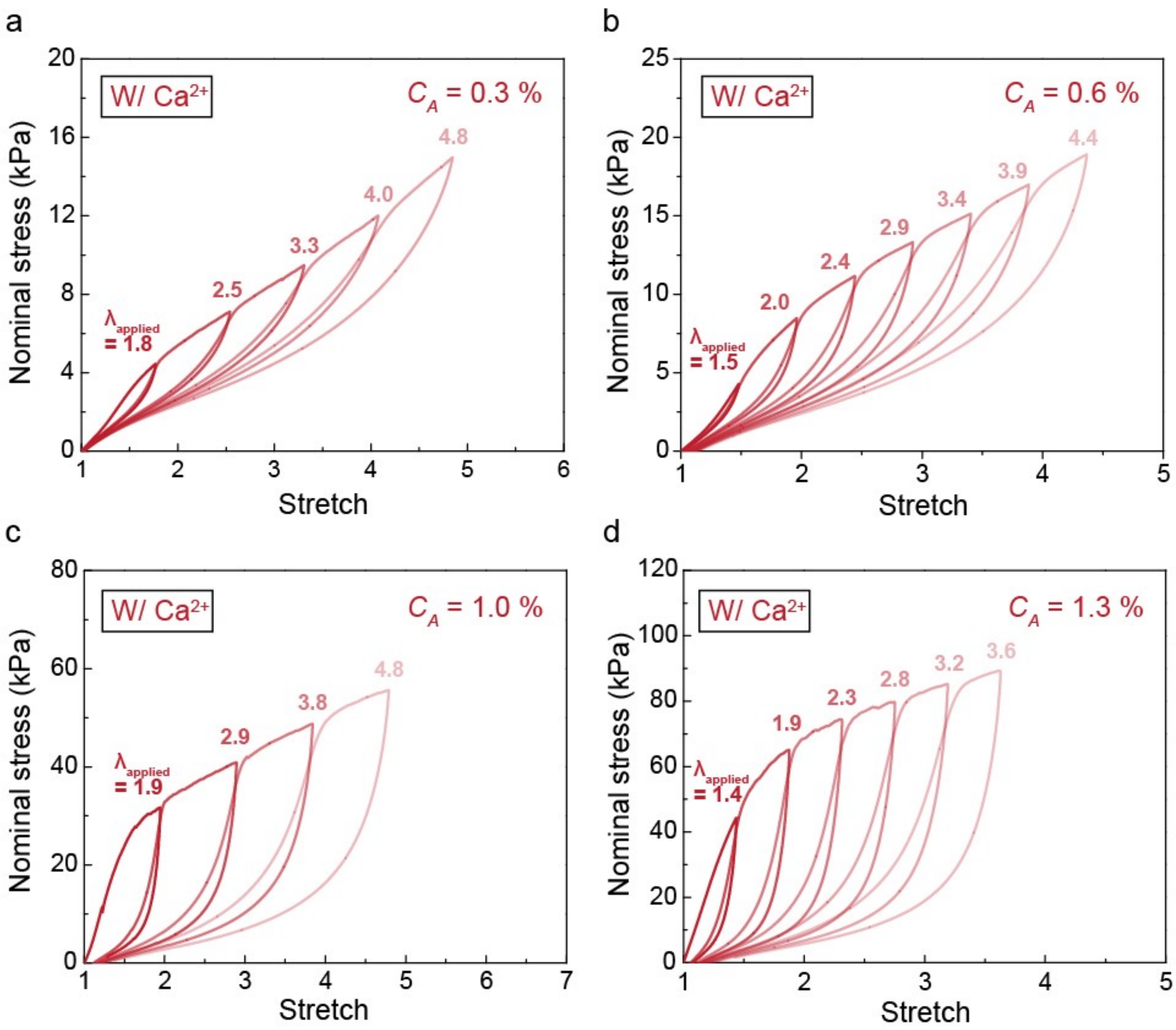

Fig. S3. Nominal stress versus stretch curves of PAAm-alginate hydrogels with Ca^{2+} under one cycle of loading and unloading. (a) C_A = 0.3 wt%. (b) C_A = 0.6 wt%. (c) C_A = 1.0 wt%. (d) C_A = 1.3 wt%.

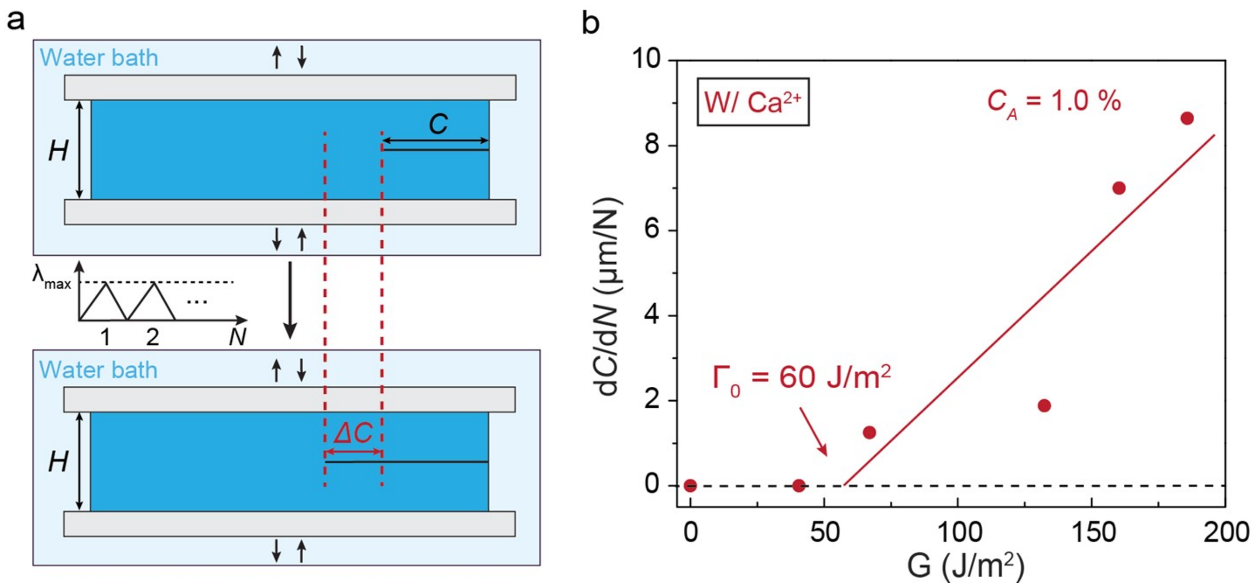

Fig. S4. Measurement of fatigue-threshold using pure-shear tensile tests. (a) Schematic illustration of the fatigue characterization using pure-shear tensile tests. (b) A representative crack extension curve in the plot of crack extension rate (i.e., d*C*/d*N*) as a function of applied energy release rate G (J/m^2). The fatigue threshold is identified as $\Gamma_0 = 60\,J/m^2$ for the hydrogel with Ca^{2+} and alginate concentration of C_A = 1.0 wt%.

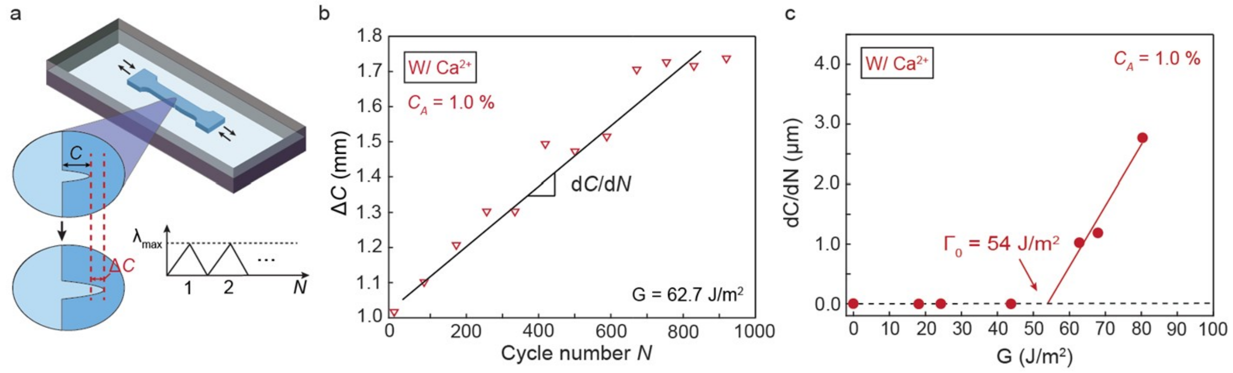

Fig. S5. Measurement of fatigue-threshold using single-notch tensile tests. (a) Schematic illustration of the fatigue characterization using single tensile tests. (b) A representative crack extension ΔC versus cycle number N for the hydrogel with Ca^{2+} and alginate concentration of C_A = 1.0 wt% at the applied energy release rate of $\mathrm{G} = 62.7\ \mathrm{J/m^2}$. (c) A representative crack extension curve in the plot of crack extension rate (i.e., dC/dN) as a function of applied energy release rate G (J/m^2). The fatigue threshold is identified as $\Gamma_0 = 54\ \mathrm{J/m^2}$ for the hydrogel with Ca^{2+} and alginate concentration of C_A = 1.0 wt%.

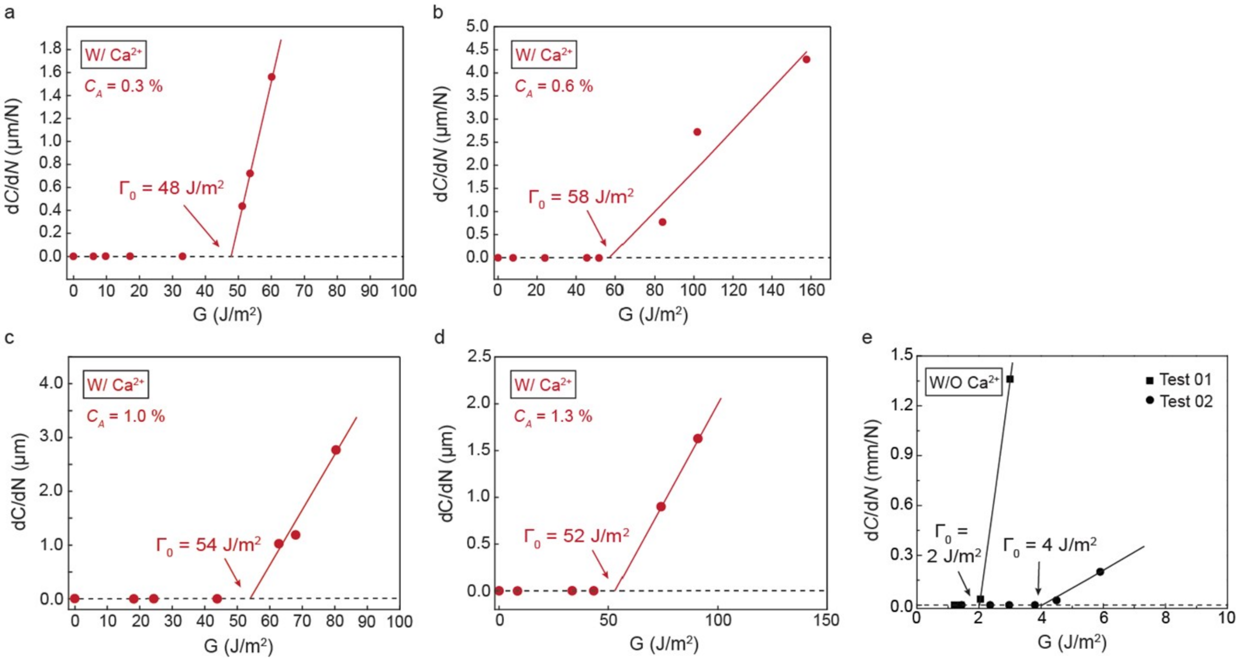

Fig. S6. Summarized fatigue-induced crack extension curves. (a) Hydrogels with Ca^{2+} and alginate concentration C_A = 0.3 wt%. (b) Hydrogels with Ca^{2+} and alginate concentration C_A = 0.6 wt%. (c) Hydrogels with Ca^{2+} and alginate concentration C_A = 1.0 wt%. (d) Hydrogels with Ca^{2+} and alginate concentration C_A = 1.3 wt%. (e) Hydrogels with no alginate or no Ca^{2+}.

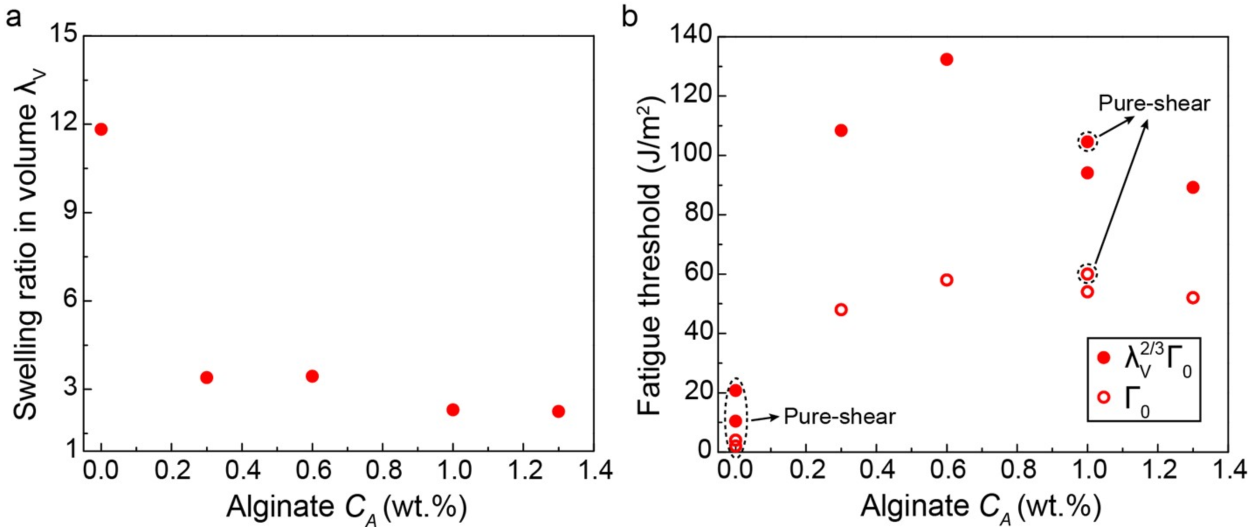

Fig. S7. (a) Swelling ratio in volume of the hydrogels λ_V with various alginate concentrations soaking in a bath of 0.01 wt% calcium chloride. (b) Summarized fatigue thresholds of hydrogels with various alginate concentrations in the swollen state Γ_0 (hollow dots) and in the as-prepared state $\lambda_V^{2/3}\Gamma_0$ (solid dots). The circled data are measured using pure-shear method and the other data are measured using single-notch method.

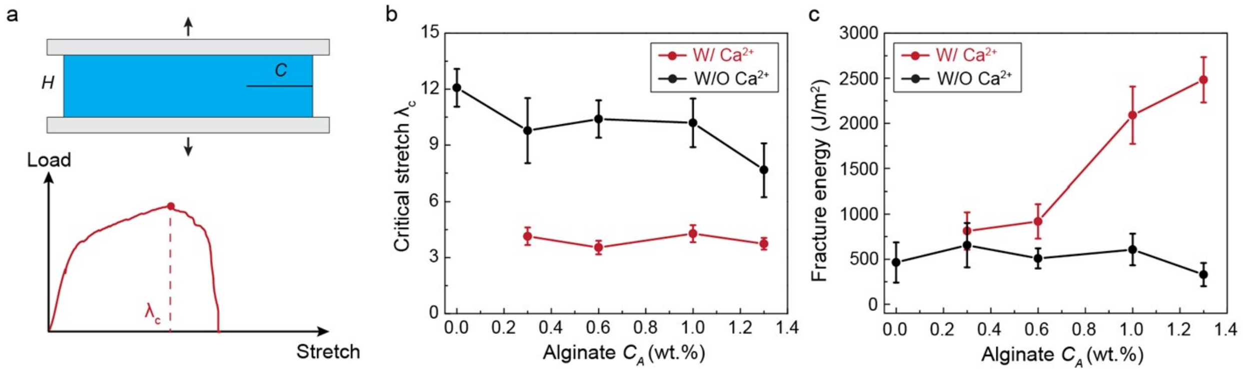

Fig. S8. Fracture test to measure the fracture toughness of the two series of PAAm-alginate hydrogels. (a) Schematic illustration of the pure shear tensile test for fracture test. (b) Summarized critical stretches. (c) Summarized fracture energies.
